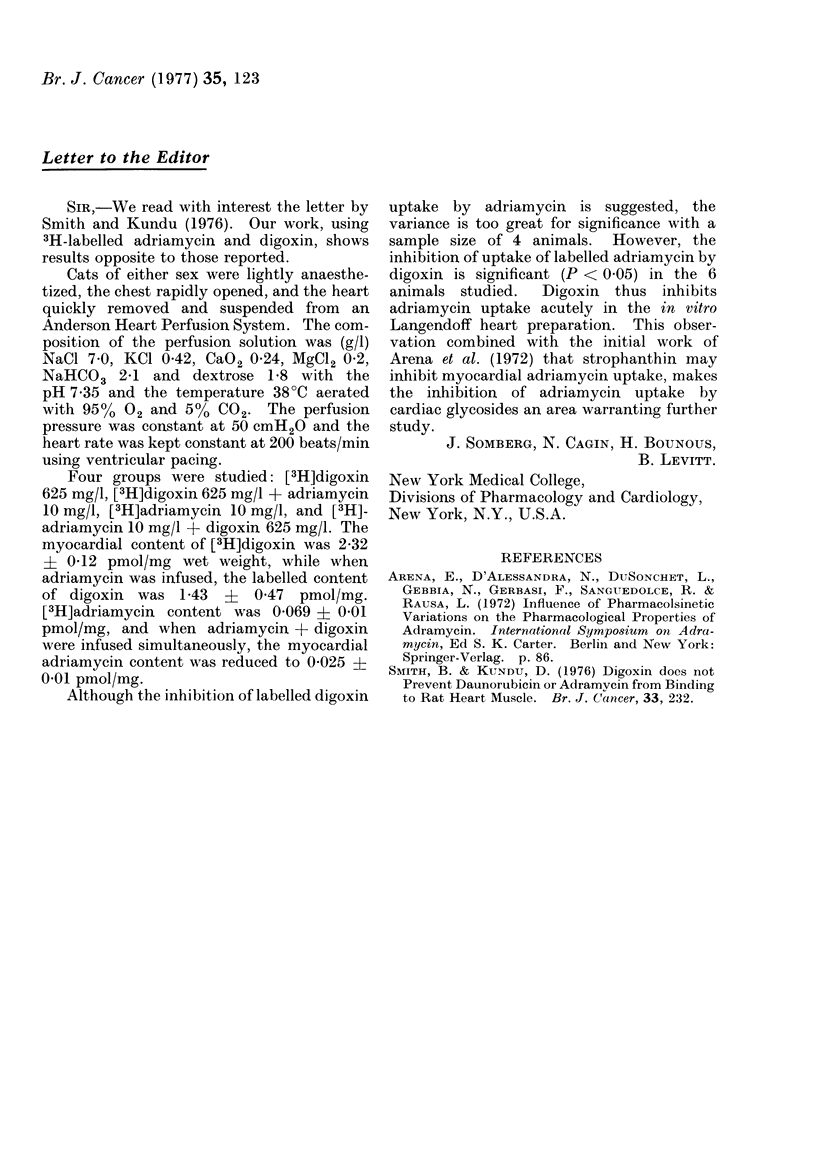# The effect of digoxin on the binding of adriamycin to cat heart muscle.

**DOI:** 10.1038/bjc.1977.13

**Published:** 1977-01

**Authors:** J. Somberg, N. Cagin, H. Bovnous, B. Levitt


*Br. J. Cancer* (1977) **35**, 123

### Letter to the Editor

SIR,—We read with interest the letter by Smith and Kundu (1976). Our work, using $^3$H-labelled adriamycin and digoxin, shows results opposite to those reported.

Cats of either sex were lightly anaesthetized, the chest rapidly opened, and the heart quickly removed and suspended from an Anderson Heart Perfusion System. The composition of the perfusion solution was (g/l) NaCl 7·0, KCl 0·42, $CaO_2$ 0·24, $MgCl_2$ 0·2, $NaHCO_3$ 2·1 and dextrose 1·8 with the pH 7·35 and the temperature 38°C aerated with 95% $O_2$ and 5% $CO_2$. The perfusion pressure was constant at 50 $cmH_2O$ and the heart rate was kept constant at 200 beats/min using ventricular pacing.

Four groups were studied: [$^3$H]digoxin 625 mg/l, [$^3$H]digoxin 625 mg/l + adriamycin 10 mg/l, [$^3$H]adriamycin 10 mg/l, and [$^3$H]-adriamycin 10 mg/l + digoxin 625 mg/l. The myocardial content of [$^3$H]digoxin was 2·32 ± 0·12 pmol/mg wet weight, while when adriamycin was infused, the labelled content of digoxin was 1·43 ± 0·47 pmol/mg. [$^3$H]adriamycin content was 0·069 ± 0·01 pmol/mg, and when adriamycin + digoxin were infused simultaneously, the myocardial adriamycin content was reduced to 0·025 ± 0·01 pmol/mg.

Although the inhibition of labelled digoxin uptake by adriamycin is suggested, the variance is too great for significance with a sample size of 4 animals. However, the inhibition of uptake of labelled adriamycin by digoxin is significant ($P < 0{\cdot}05$) in the 6 animals studied. Digoxin thus inhibits adriamycin uptake acutely in the *in vitro* Langendoff heart preparation. This observation combined with the initial work of Arena *et al.* (1972) that strophanthin may inhibit myocardial adriamycin uptake, makes the inhibition of adriamycin uptake by cardiac glycosides an area warranting further study.

J. SOMBERG, N. CAGIN, H. BOUNOUS, B. LEVITT.

New York Medical College,
Divisions of Pharmacology and Cardiology,
New York, N.Y., U.S.A.

REFERENCES

ARENA, E., D'ALESSANDRA, N., DUSONCHET, L., GEBBIA, N., GERBASI, F., SANGUEDOLCE, R. & RAUSA, L. (1972) Influence of Pharmacolsinetic Variations on the Pharmacological Properties of Adramycin. *International Symposium on Adramycin*, Ed S. K. Carter. Berlin and New York: Springer-Verlag. p. 86.

SMITH, B. & KUNDU, D. (1976) Digoxin does not Prevent Daunorubicin or Adramycin from Binding to Rat Heart Muscle. *Br. J. Cancer*, **33**, 232.